# Supplementary material for: Arioc: high-throughput read alignment with GPU-accelerated exploration of the seed-and-extend search space
Source: PeerJ. 2015 Mar 3;3:e808. doi: 10.7717/peerj.808 (PMC4358639; doi:10.7717/peerj.808)
Supplement: Supplemental Information 1 [file peerj-03-808-s001.pdf]

Supplementary Results

Arioc: high-throughput read alignment with GPU-accelerated exploration of the seed-and-extend search space

Richard Wilton, Tamas Budavari, Ben Langmead, Sarah Wheelan, Steven L. Salzberg, and Alex Szalay

Tables

Table T1 Candidates for performance comparisons.
Table T2 Software versions.
Table T3 Software configuration parameters.
Table T4 Distance between simulated and reported mapping positions.

Results for simulated unpaired reads

Figure S1 Correctly mapped versus incorrectly mapped reads, for 1 million simulated 100nt unpaired Illumina reads. Empirical error rate 0.9%.

Figure S2 Correctly mapped versus incorrectly mapped reads, for 1 million simulated 100nt unpaired Illumina reads. Empirical error rate 1.4%.

Figure S3 Correctly mapped versus incorrectly mapped reads, for 1 million simulated 250nt unpaired Illumina reads. Empirical error rate 0.9%.

Figure S4 Correctly mapped versus incorrectly mapped reads, for 1 million simulated 250nt unpaired Illumina reads. Empirical error rate 1.5%.

Results for simulated paired-end reads

Figure S5 Correctly mapped versus incorrectly mapped reads, for 1 million simulated 100nt paired-end Illumina reads (2 million total reads). Empirical error rate 0.9%.

Figure S6 Correctly mapped versus incorrectly mapped reads, for 1 million simulated 100nt paired-end Illumina reads (2 million total reads). Empirical error rate 1.4%.

Figure S7 Correctly mapped versus incorrectly mapped reads, for 1 million simulated 250nt paired-end Illumina reads (2 million total reads). Empirical error rate 0.9%.

Figure S8 Correctly mapped versus incorrectly mapped reads, for 1 million simulated 250nt paired-end Illumina reads (2 million total reads). Empirical error rate 1.5%.

Results for Illumina 100nt unpaired reads

Figure S9 Throughput versus sensitivity, for 10 million 100nt unpaired reads from the YanHuang genome.

Additional results for Illumina 100nt paired-end reads

Figure S10 Throughput versus sensitivity. (With additional results for an edit-distance aligner).

Figure S11 Correctly mapped versus incorrectly mapped reads, for 1 million simulated 100nt paired-end Illumina reads (2 million total reads). Empirical error rate 4.3%. (Including results for an edit-distance aligner.)

BWA-MEM	Li 2013	CPU	approximately 45000 reads/sec (Figure 6)
Bowtie 2	Langmead & Salzberg 2012	CPU	approximately 12000 reads/sec (Figure 6)
NVBIO	NVidia 2014	GPU	approximately 60000 reads/sec (Figure 6)
SOAP3-dp	Luo 2013	GPU	approximately 100000 reads/sec (Figure 6)
SNAP	Zaharia et al 2011	CPU	does not compute Smith-Waterman alignments
RazerS 3	Weese et al 2012	CPU	does not compute Smith-Waterman alignments; 3x slower than BWA
YARA	Siragusa et al 2014	CPU	does not compute Smith-Waterman alignments
GraBFAST	Narang et al 2012	GPU	1455 reads/sec (8 million reads / 2749 seconds / 2 GPUs)
BarraCUDA	Klus et al 2012	GPU	17000 reads/sec (14 million 76nt pairs / 27 minutes)
BWT-GPU	Torres et al 2012	GPU	exact alignments only (does not compute local alignments)
CUSHAW2-GPU	Liu & Schmidt 2014	GPU	1.3x speedup versus 12-threaded Bowtie 2
PEANUT	Köster & Rahmann 2014	GPU	2-3x speedup versus 8-threaded BWA-MEM

Table T1. Candidates for performance comparisons. We considered the above CPU-based and GPU-based read aligners for detailed speed and sensitivity comparisons.

We carried out detailed performance comparisons only with aligners that perform Smith-Waterman local alignment, that can handle both unpaired and paired-end mapping, and that are capable of computing alignments on a large number (hundreds of millions) of short (100nt-250nt) reads on a single computer.

We excluded aligners whose speed was not at least twice that of BWA or Bowtie on 24 CPU threads with comparable sensitivity (see Figure S10), or for which other practical considerations precluded a direct comparison using both simulated and sequencer-generated datasets.

In particular, we excluded aligners that use Levenshtein edit distance rather than Smith-Waterman dynamic programming as a similarity metric because such aligners discover and report mappings that would be rejected by Smith-Waterman aligners. This occurs because there is no explicit model for insertions and deletions (indels) in the computation of edit distance. Consequently, edit-distance aligners and Smith-Waterman aligners behave differently in regard to reads with indels; this can be demonstrated by using a read simulator to generate reads with a higher probability of indels (see Figure S11).

This phenomenon also distorts speed-versus-sensitivity comparisons between edit-distance aligners and Smith-Waterman aligners. All read aligners limit the number of alignments they compute by abandoning the search for additional mappings for a read when they have discovered a sufficient number of high-scoring mappings for that read. Because edit-distance aligners assign high scores for some gapped mappings that would have low Smith-Waterman alignment scores, they may prematurely abandon the search for additional mappings for a read before they discover a mapping with fewer indels for that read. This is illustrated in Figure S10, in which Smith-Waterman scores are computed for all reported mappings: a significant number of mappings reported by edit-distance aligners have Smith-Waterman scores that do not meet a minimum threshold score.

A read aligner can obtain high throughput by using a linear-time edit-distance computation instead of a polynomial-time Smith-Waterman computation, but the concomitant loss in fidelity invalidates direct performance comparisons with Smith-Waterman aligners except for reads that contain few or no indels.

References for Table T1.

Klus P et al. (2012) BarraCUDA – a fast short read sequence aligner using graphics processing units. BMC Research Notes **5**:27.

Köster J and Rahmann S. (2014) Massively parallel read mapping on GPUs with the q-group index and PEANUT. PeerJ 2:e606.

Langmead B and Salzberg S. (2012) Fast gapped-read alignment with Bowtie 2. Nature Methods **9**, 357-359.

Li H. (2013) Aligning sequence reads, clone sequences and assembly contigs with BWA-MEM. arXiv 1303.3997v1.

Liu Y and Schmidt B. (2014) CUSHAW2-GPU: empowering faster gapped short-read alignment using GPU computing. IEEE Design & Test **31**:1, 31-39.

Luo R et al. (2013) SOAP3-dp: Fast, Accurate and Sensitive GPU-Based Short Read Aligner. PLOS ONE **8**:5.

Narang A et al. (2012) GrABFAST: a CUDA based GPU accelerated fast short sequence alignment algorithm. *20th Annual Conference on High Performance Computing (HiPC)*, 1-9.

NVidia Corporation. (2014) NVBIO. http://nvlabs.github.io/nvbio, downloaded October 2014.

Siragusa E et al. (2014) Yara — Yet another read aligner. http://www.seqan.de/projects/yara, downloaded November 2014.

Torres JS et al. (2012) Using GPUs for the exact alignment of short-read genetic sequences by means of the Burrows-Wheeler transform. IEEE Comp Bio **9**:4, 1245-1256.

Weese D et al. (2012) RazerS: faster, fully sensitive read mapping. Bioinformatics **28**:20, 2592-2599.

Zaharia M et al. (2011) Faster and more accurate sequence alignment with SNAP. arXiv:1111.5572 [cs.DS]

Bowtie 2	2.1.0 (64-bit)
BWA-MEM	0.7.9a-r786
SOAP3-dp	2.3.178
NVBIO	0.9.98
SNAP	1.0beta.14

Table T2. Software versions. All binaries executed using Red Hat Scientific Linux release 5.10 and CUDA v6.5.

	High throughput	High sensitivity
Arioc	`<gapped maxJ="16" seedDepth="2" />`	`<gapped maxJ="1024" seedDepth="6" />`
Bowtie 2	`-D5`<br>`-R3`<br>`-I C,3,0`<br>`--ignore-quals`	`-D200`<br>`-R3`<br>`-I C,3,0`<br>`--ignore-quals`
BWA-MEM	`-r3.0`<br>`-I 450,50,500,1`	`-r1.05`<br>`-I 450,50,500,1`
SOAP3-dp	`NumOfCpuThreads=16`<br>`MaxHitsEachEndForPairing=8000`<br>`Soap3MisMatchAllow=2`	`NumOfCpuThreads=16`<br>`MaxHitsEachEndForPairing=32000`<br>`Soap3MisMatchAllow=4`
NVBIO	`-D 4`<br>`--min-ext 2`<br>`--max-ext 4`<br>`--seed-freq S,20,0`<br>`--max-reseed 0`	`-D 500`
SNAP	`-d 15`<br>`-h 300`	`-d 45`<br>`-n 81`<br>`-h 1024`

Table T3. Software configuration parameters. Non-default parameters for the two extreme data points in Figure 6 (speed versus sensitivity). All aligners were configured to perform local alignment using 20nt seeds (except BWA-MEM, for which 19nt seeds were used). For Arioc, maxJ specifies the maximum size of a "bucket" in the seed-and-extend hash table; seedDepth limits the number of seed iterations.

D	Arioc	Bowtie 2	BWA-MEM	SOAP3-dp	NVBIO
0	976039	969745	972596	922048	973572
1	1800	1682	2139	826	1868
2	23	25	26	18	23
3	7	6	6	5	7
4	5	6	6	6	6
5	3	6	7	2	7
6	0	1	1	1	1
7	1	1	0	0	0
8	0	0	0	0	0
9	0	0	0	0	0
10 to 19	0	1	0	2	1
20 to 29	7	17	14	14	12
30 to 39	16	31	19	28	14
40 to 49	3	12	12	33	11
50 to 59	7	16	12	16	14
60 to 69	5	21	10	13	11
70 to 79	1	8	9	9	8
80 to 89	7	13	11	10	10
90 to 99	8	7	10	14	13
100+	18233	28323	25080	36422	24236

Table T4. Number of reads with distance D between simulated and reported mapping positions for 1 million simulated 100nt unpaired Illumina reads.

For each mapping reported by each aligner, we used the POS and CIGAR fields in the SAM record to compute a distance metric that represented the read's best-case distance from Mason's simulated mapping:

Extract the following from the POS and CIGAR fields:

$Pstart_M$	POS reported by Mason
$Pstart_A$	POS reported by the aligner
$Cstart_M$	soft-clipped positions at the start of CIGAR, reported by Mason (always zero)
$Cstart_A$	soft-clipped positions at the start of CIGAR, reported by the aligner
S_M	total reference positions spanned by the mapping, from CIGAR reported by Mason
S_A	total reference positions spanned by the mapping, from CIGAR reported by the aligner

Compute:

$$Pstart_{Madj} = Pstart_M - Cstart_M$$
$$Pstart_{Aadj} = Pstart_A - Cstart_A$$
$$Pend_{Madj} = Pstart_{Madj} + S_M$$
$$Pend_{Aadj} = Pstart_{Aadj} + S_A$$

$$D = \min(\ \mathrm{abs}(Pstart_{Madj} - Pstart_{Aadj}), \mathrm{abs}(Pend_{Madj} - Pend_{Aadj})\ )$$

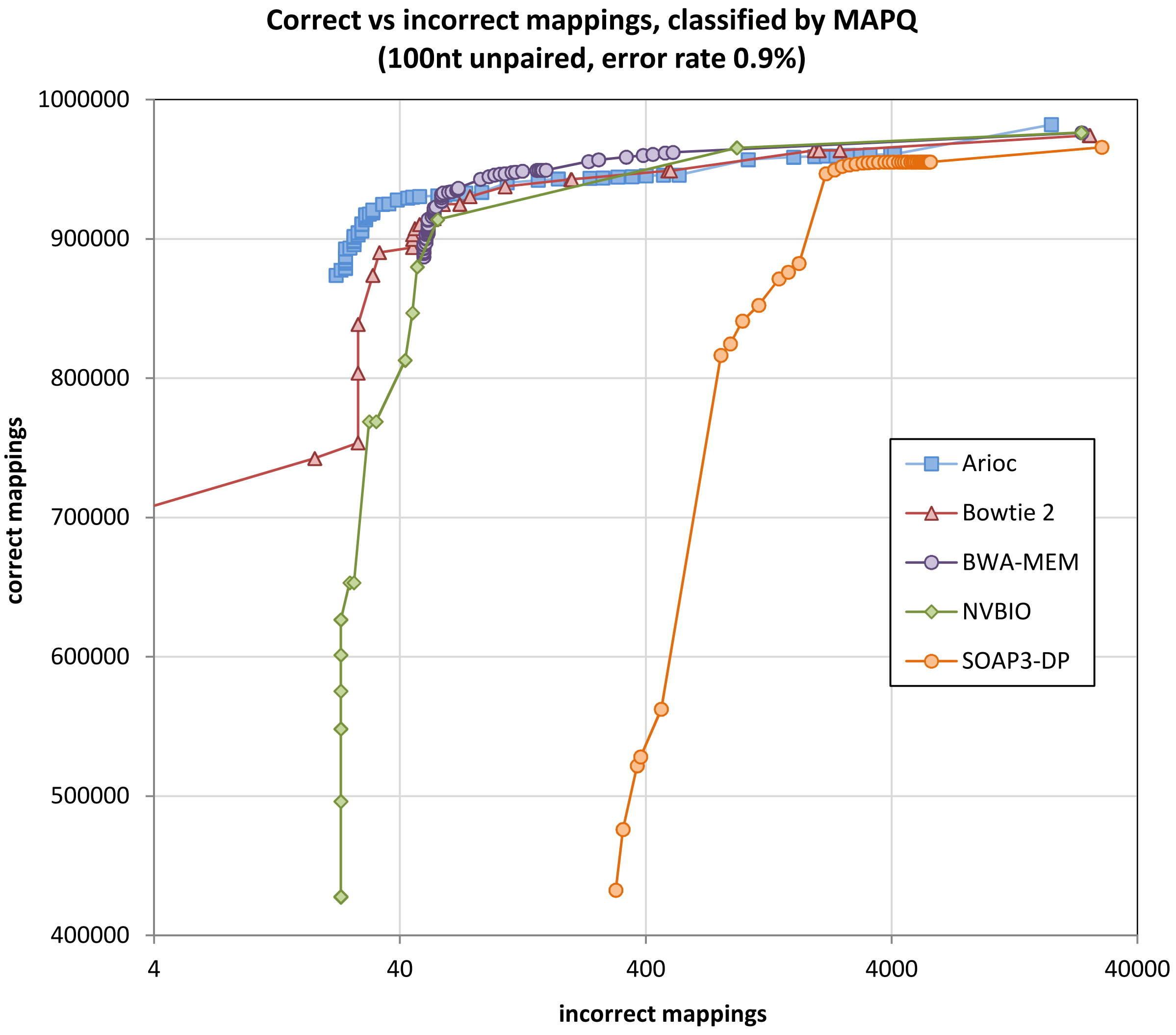

Figure S1. Total correctly mapped versus incorrectly mapped reads, plotted for decreasing MAPQ, for 1 million simulated 100nt unpaired Illumina reads.

Empirical error rate: 0.9% (Mason parameters: -hn 2 -pmm 0.004; -pi 0.001; -pd 0.001).

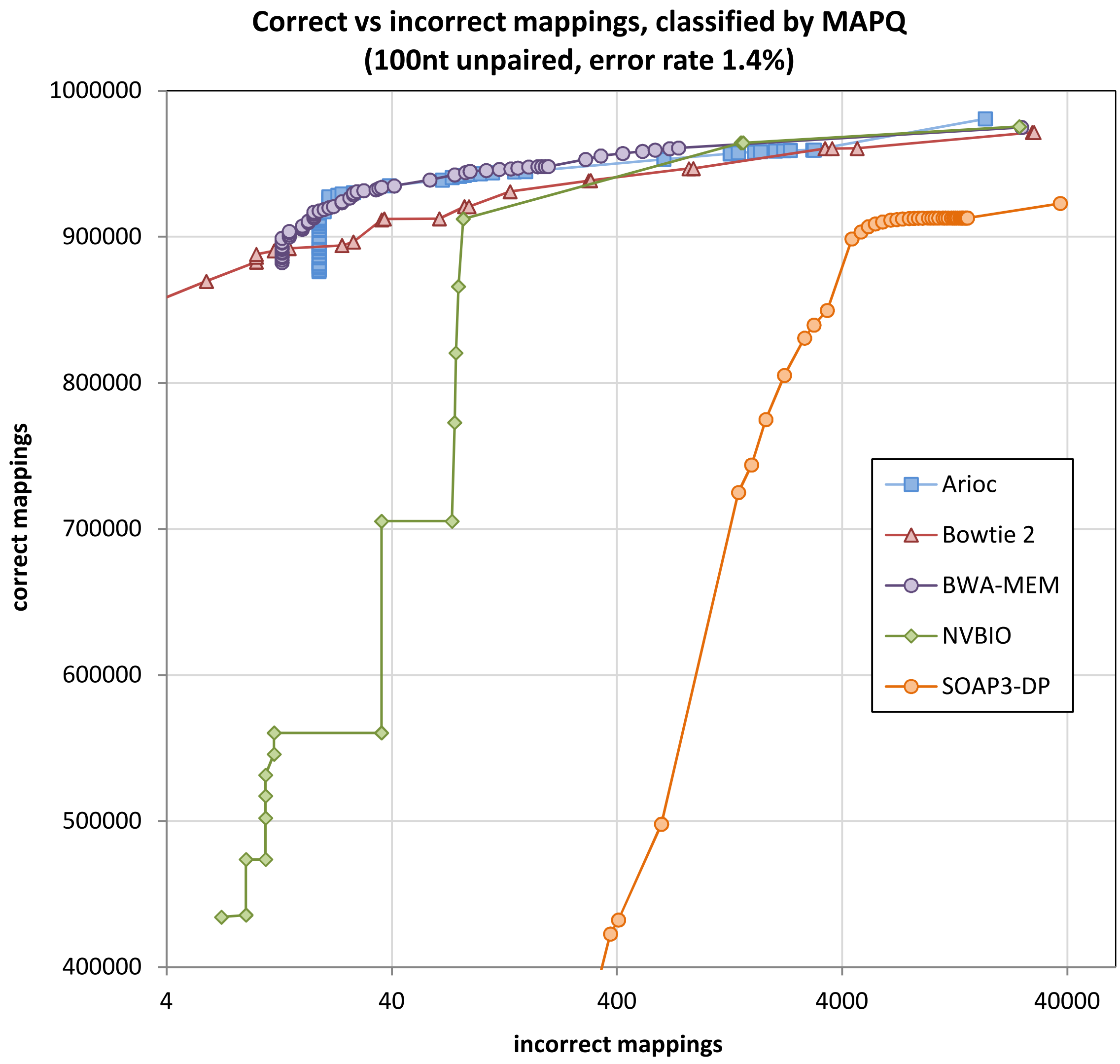

Figure S2. Total correctly mapped versus incorrectly mapped reads, plotted for decreasing MAPQ, for 1 million simulated 100nt unpaired Illumina reads.

Empirical error rate 1.4% (Mason parameters: -hn 2 -pmm 0.004; -pi 0.004; -pd 0.004).

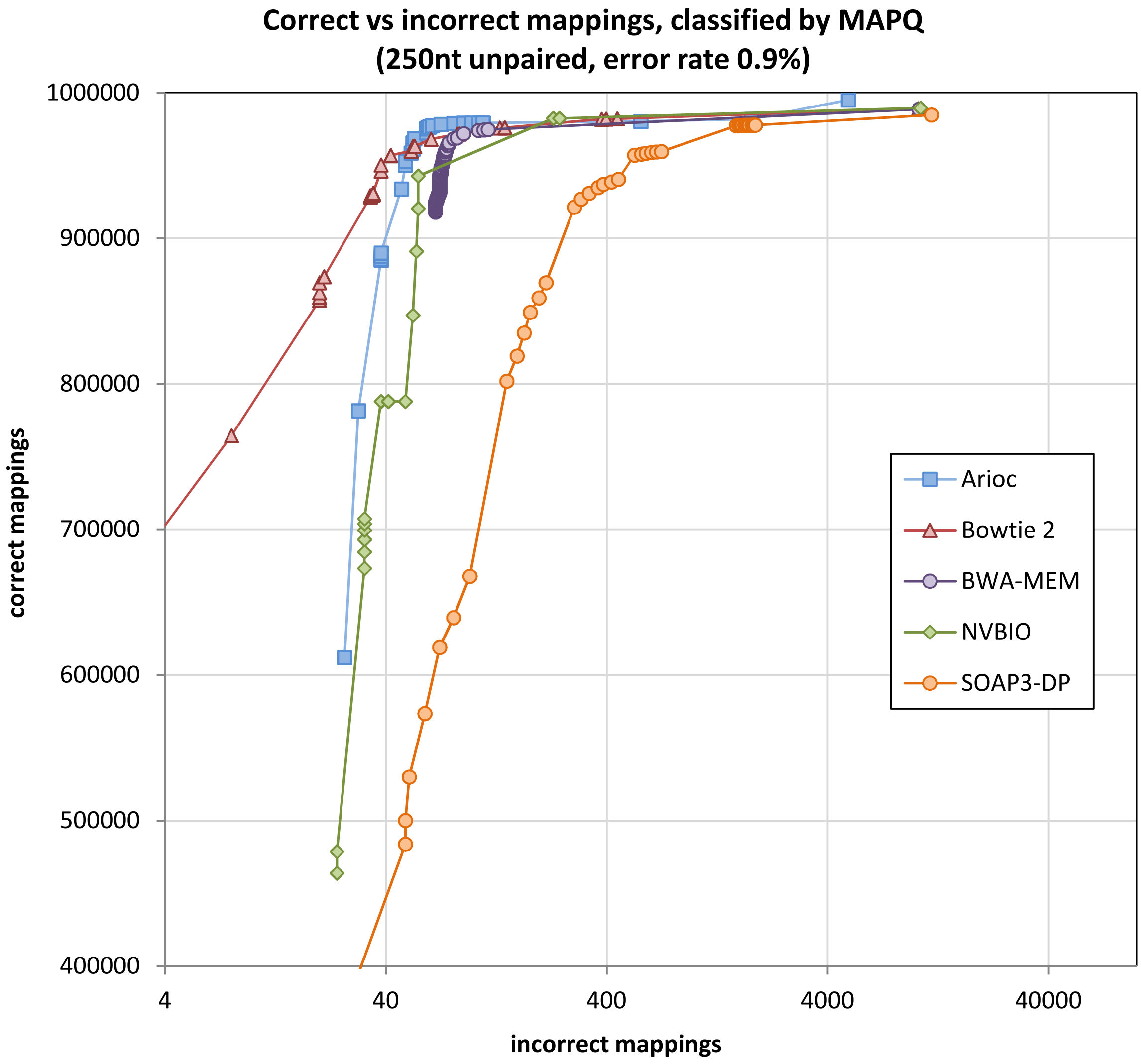

Figure S3. Total correctly mapped versus incorrectly mapped reads, plotted for decreasing MAPQ, for 1 million simulated 250nt unpaired Illumina reads.

Empirical error rate 0.9% (Mason parameters: -hn 2 -pmm 0.004; -pi 0.001; -pd 0.001).

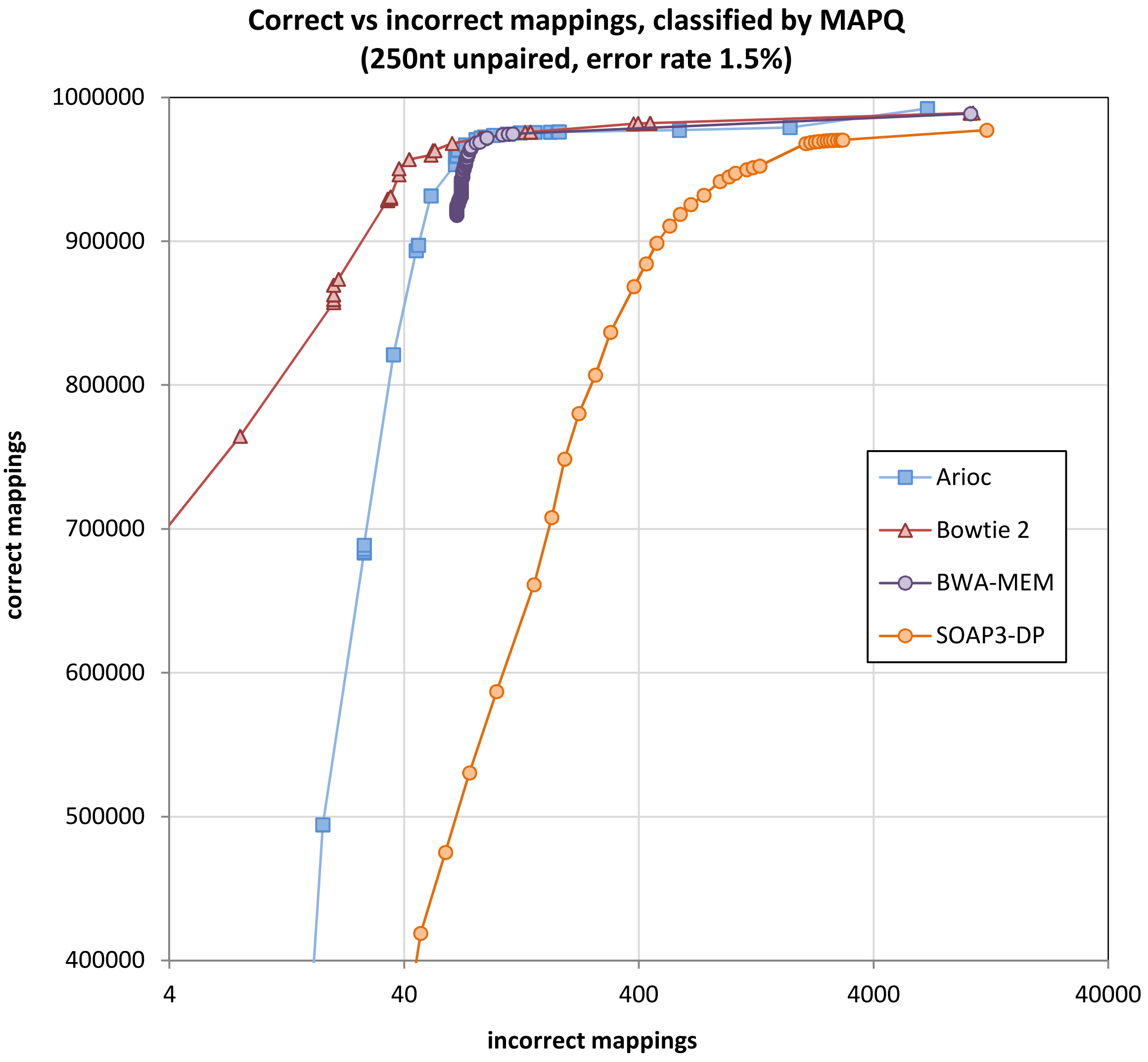

Figure S4. Total correctly mapped versus incorrectly mapped reads, plotted for decreasing MAPQ, for 1 million simulated 250nt unpaired Illumina reads.

Empirical error rate 1.5% (Mason parameters: -hn 2 -pmm 0.004; -pi 0.004; -pd 0.004).

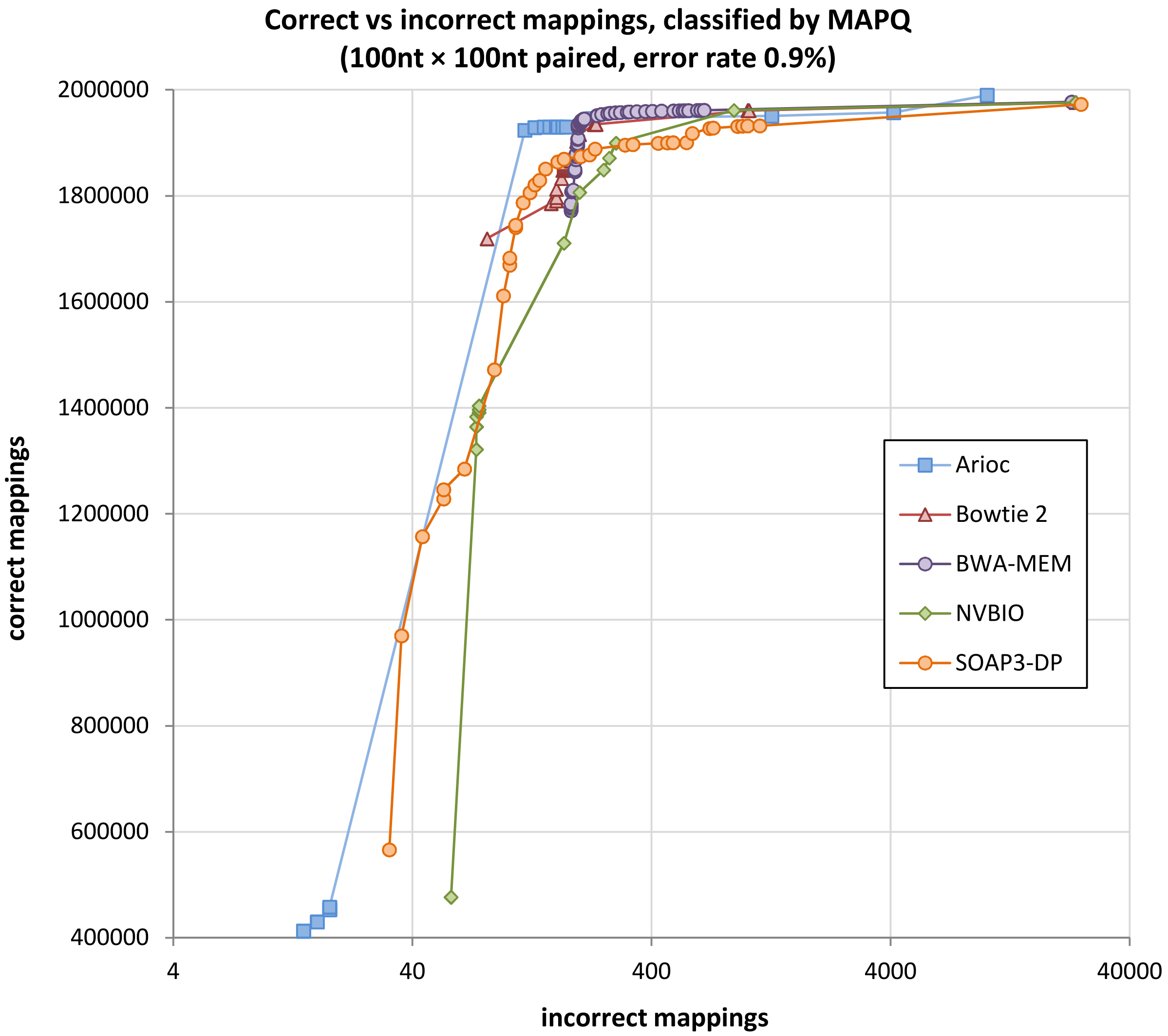

Figure S5. Total correctly mapped versus incorrectly mapped reads, plotted for decreasing MAPQ, for 1 million simulated 100nt paired-end Illumina reads (2 million total reads).

Empirical error rate 0.9% (Mason parameters: -hn 2 -pmm 0.004; -pi 0.001; -pd 0.001).

(Same as Figure 7.)

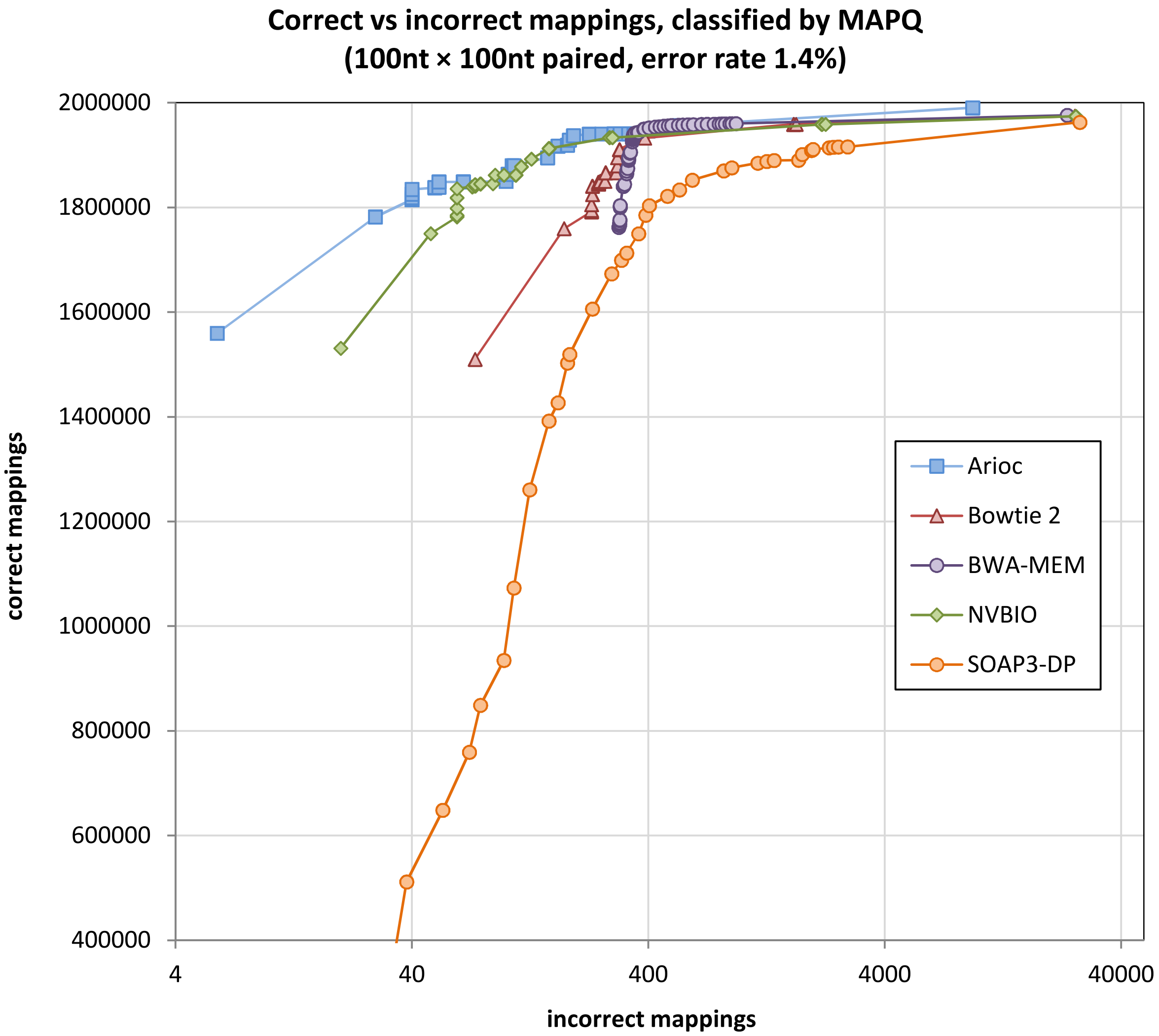

Figure S6. Total correctly mapped versus incorrectly mapped reads, plotted for decreasing MAPQ, for 1 million simulated 100nt paired-end Illumina reads (2 million total reads).

Empirical error rate 1.4% (Mason parameters: -hn 2 -pmm 0.004; -pi 0.004; -pd 0.004).

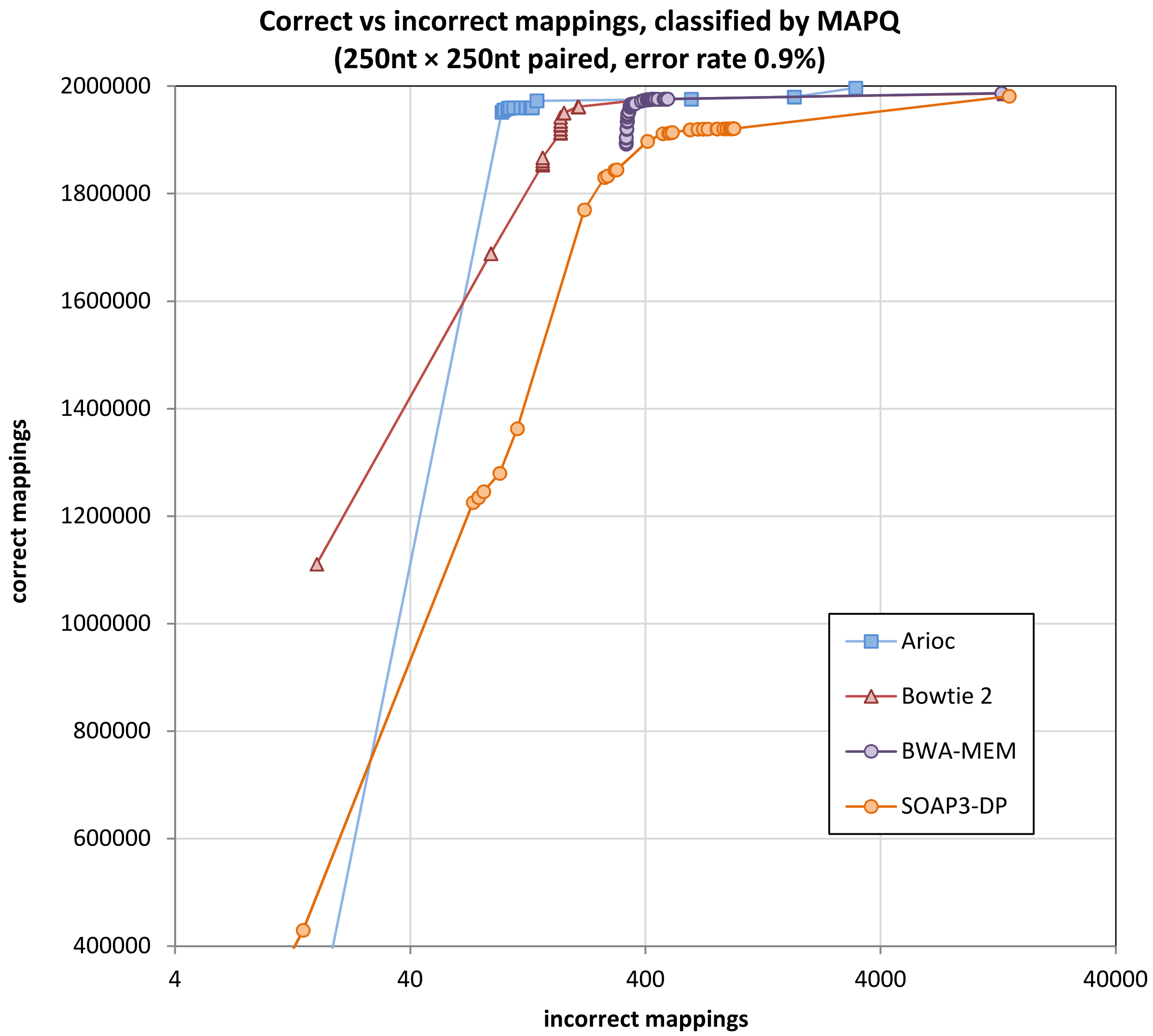

Figure S7. Total correctly mapped versus incorrectly mapped reads, plotted for decreasing MAPQ, for 1 million simulated 250nt paired-end Illumina reads (2 million total reads).

Empirical error rate 0.9% (Mason parameters: -hn 2 -pmm 0.004; -pi 0.001; -pd 0.001).

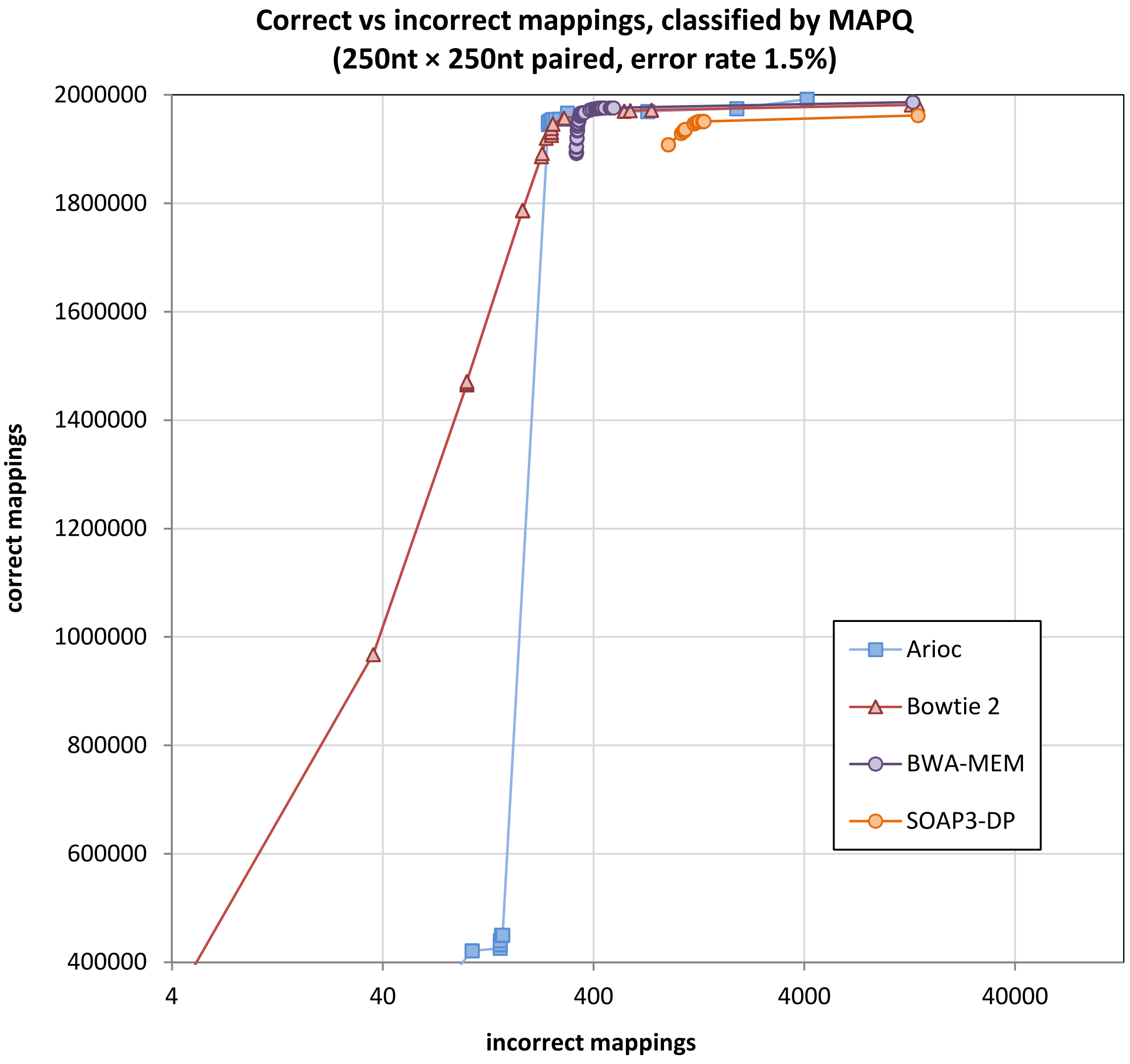

Figure S8. Total correctly mapped versus incorrectly mapped reads, plotted for decreasing MAPQ, for 1 million simulated 250nt paired-end Illumina reads (2 million total reads).

Empirical error rate 1.5% (Mason parameters: -hn 2 -pmm 0.004; -pi 0.004; -pd 0.004).

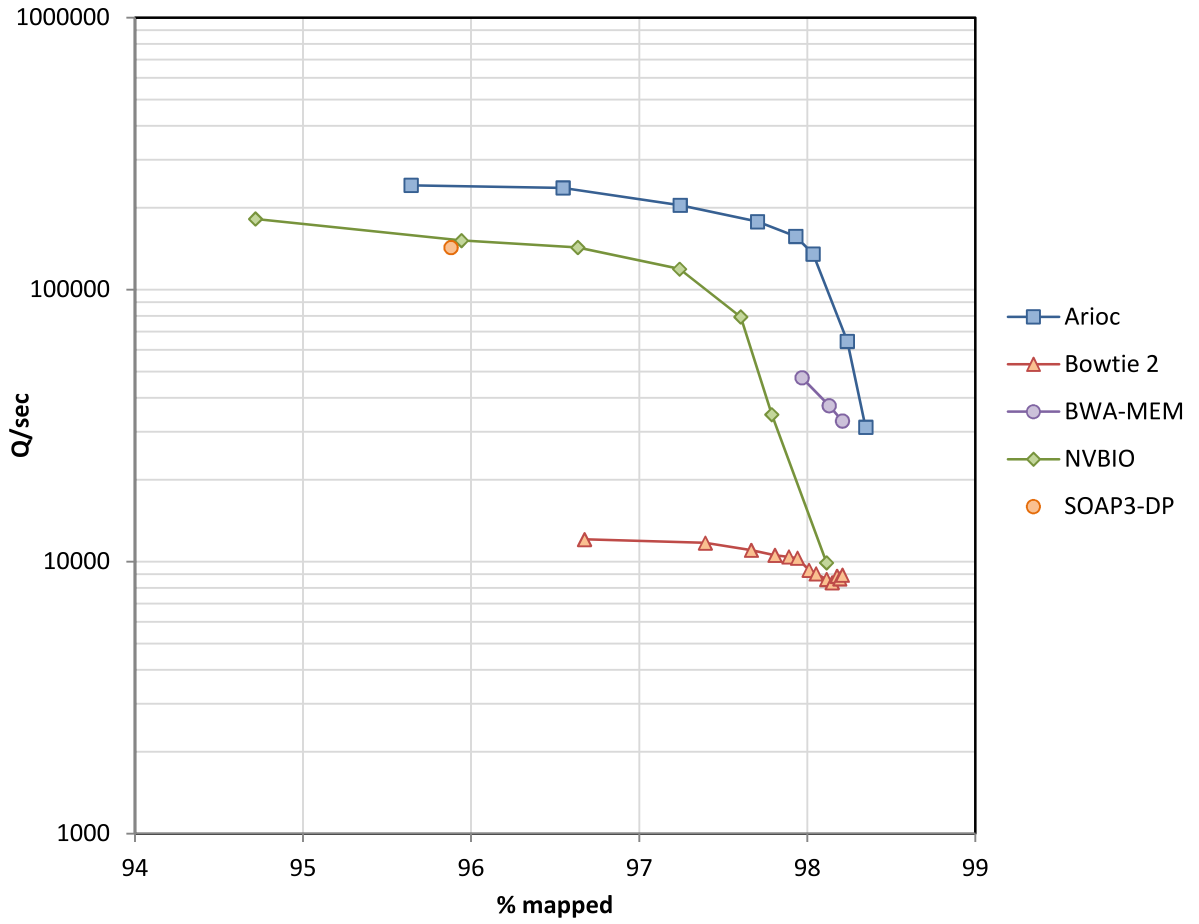

Figure S9. Throughput (measured as the number of 100nt query sequences processed per second) plotted versus sensitivity (expressed as the percentage of mapped reads). Data for 10 million 100nt unpaired reads from the YanHuang genome. Workstation hardware: 12 CPU cores (24 threads of execution), one NVidia K20c GPU.

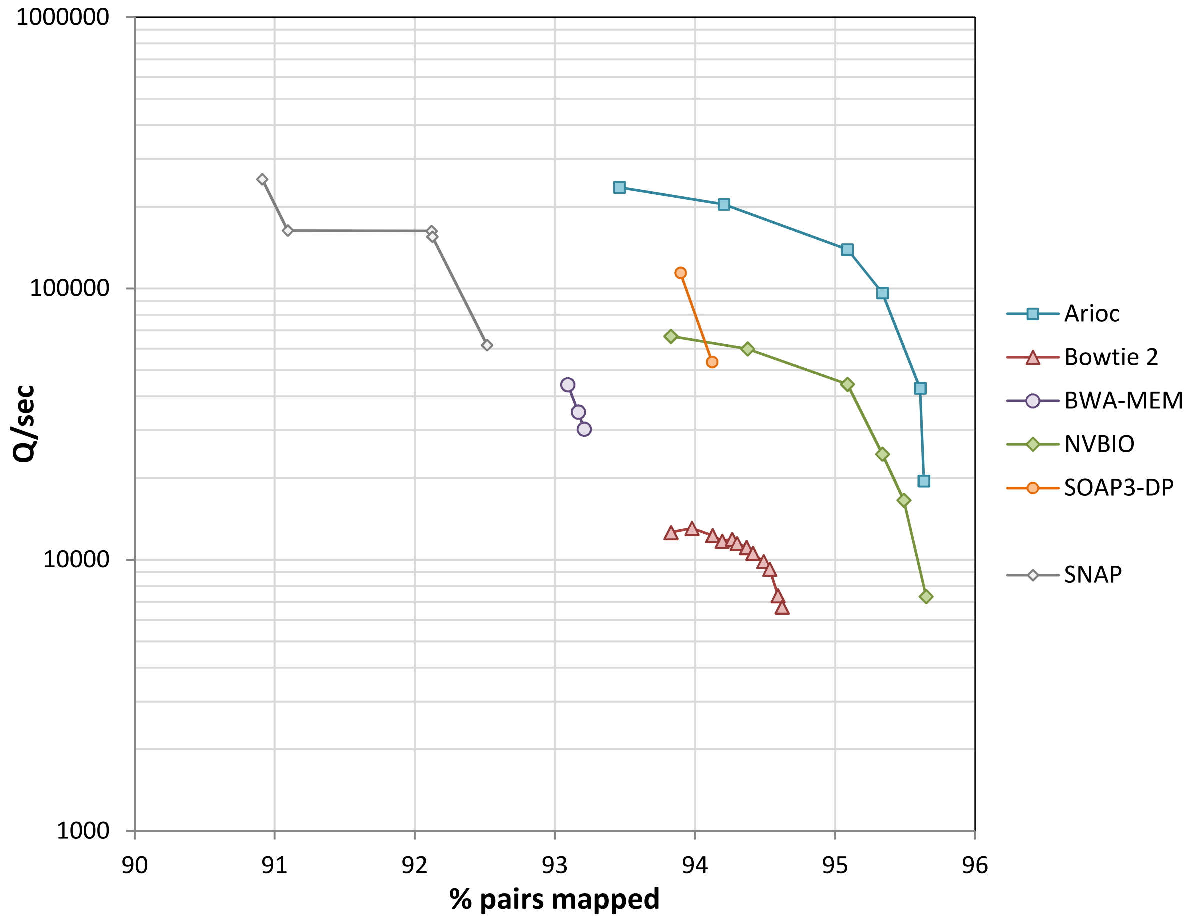

Figure S10. Throughput versus sensitivity for aligners. Same data as Figure 8, with additional results for SNAP and YARA on 24 CPU threads.

Results for YARA (not plotted): 2100 Q/sec for 85% pairs mapped.

A pair was considered to be "mapped" when the following conditions were met:

- Alignment score >= 100 for both mates using the following scoring parameters: match=+2; mismatch=−6; gap open=−5; gap space=−3.
- Mates in expected orientation (forward-reverse).
- Fragment length between 1 and 500.

(See also Table T1.)

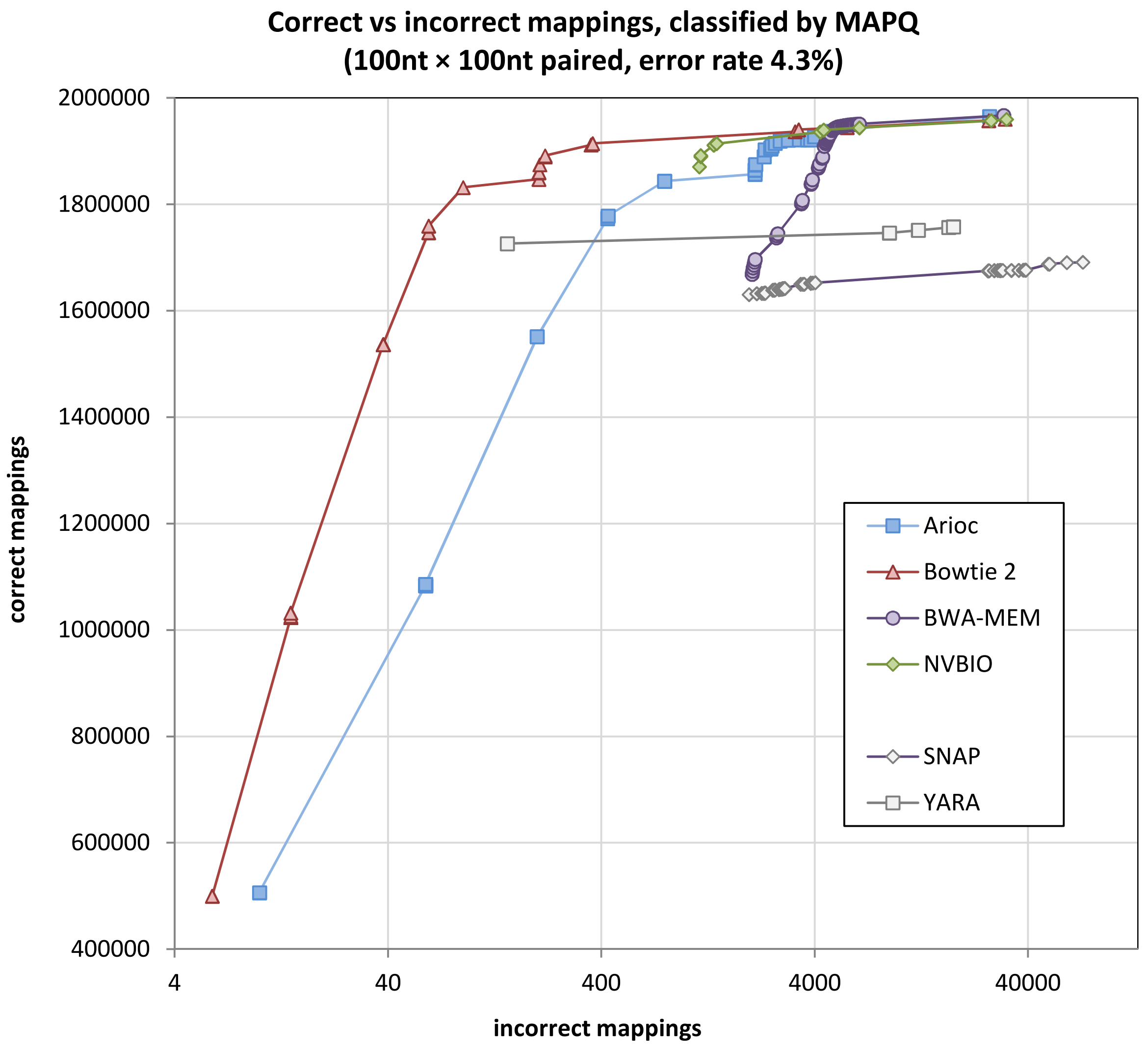

Figure S11. Total correctly mapped versus incorrectly mapped reads, plotted for decreasing MAPQ, for 1 million simulated 100nt paired-end Illumina reads (2 million total reads), with additional results for edit-distance aligners SNAP and YARA.

Empirical error rate 4.3% (Mason parameters: -hn 2 -pmm 0.015; -pi 0.015; -pd 0.015).

(See also Table T1.)
